# Description of a novel *Ligia* species from Nihoa, a remote island in the Papahānaumokuākea Marine National Monument

**DOI:** 10.7717/peerj.19373

**Published:** 2025-05-22

**Authors:** Carlos A. Santamaria, Annabelle Bork, Alexandra J. Larson, Daniel J. Link

**Affiliations:** 1 Department of Biology, University of Tampa, Tampa, FL, United States of America; 2 Papahānaumokuākea Marine National Monument, US Fish and Wildlife Service, Honolulu, Hawai‘ i, United States of America

**Keywords:** Oniscidea, Cryptic species, Ligiidae, Intertidal, Species description, Pacific biodiversity

## Abstract

Isopods in the genus *Ligia* have been shown to harbor deeply divergent genetic lineages that have, in some instances, been recognized as cryptic species. For instance, the use of molecular taxonomic approaches to characterize coastal *Ligia* from the Hawaiian Islands led to the redescription of *Ligia hawaiensis*, the sole endemic coastal species previously recognized in the region, and to the description of seven new species endemic to the region. These species appear to be highly restricted to rift zones within single islands, single islands, or previously connected islands, suggesting these species evolved in allopatry. These findings, coupled with the poor dispersal capabilities exhibited by *Ligia* isopods and the geology of the Hawaiian Islands, suggest that additional cryptic species may exist in highly isolated populations yet to be studied. Studies to date have characterized *Ligia* from throughout the younger Hawaiian Islands (*e.g.*, Kaua‘ i, O‘ ahu, Moloka‘ i, Maui, Lanai, and Hawai‘ i); however, no endemic *Ligia* populations from the older islands and more remote islands that form part of the Papahānaumokuākea Marine National Monument (PMNM) have been studied. This region represents the largest marine conservation area in the USA, and includes at least three islands where *L. hawaiensis* have been previously reported from. Herein, we apply molecular taxonomic approaches to characterize *Ligia* specimens from Nihoa, a remote island in the PMNM. Results show that *Ligia* from Nihoa form a highly divergent that is reciprocally monophyletic lineage with other Hawaiian *Ligia* species. This lineage, described as *Ligia barack* sp. nov., adds to the known biodiversity of the PMNM and highlights the importance of continued exploration and conservation of this remote and highly biodiverse region.

## Introduction

The Hawaiian Islands (hereafter HI) are a series of islands, atolls, islets, and rocky outcroppings of volcanic origin spanning ∼2,400-km of the northern Pacific Ocean. Islands in the archipelago are arranged in a relatively linear manner, with younger islands located towards the eastern end of the archipelago and older islands found in its western end. Younger islands include eight major islands, all of which have formed in the past 5 million years (in decreasing age: Ni‘ ihau, Kaua‘ i, O‘ ahu, Moloka‘ i, Maui, Lanai, Kaho‘ olawe, and Hawai‘ i). The older islands, found west of Kaua‘ i, include ten island groups ranging widely in size and elevation (in decreasing age: Kure Atoll, Midway Atoll, Pearl & Hermes Atoll, Lisianski, Laysan, Maro Reef, Gardner Pinnacles, French Frigate Shoals, Necker, and Nihoa). These islands are part of the Papahānaumokuākea Marine National Monument (hereafter “PMNM”), a protected area of the United States of America established by presidential decree on June 15, 2006 to protect natural and cultural resources from the region. The Monument initially protected 362,073 km^2^ of marine habitats; however, it was extended by President Barack H. Obama in 2016 to encompass 1,508,870 km^2^ of the Pacific Ocean. This makes the PMNM the largest contiguous fully protected conservation in the United States of America and one of the largest marine preserves in the world. The habitats of the PMNM support an incredible diversity of coral, fish, birds, marine mammals and other flora and fauna, many of which are endemic to the PMNM (Starr & Martz, 1999; Starr & Starr, 2008; Kane, Kosaki & Wagner, 2014). Nonetheless, recent descriptions of new species from the PMNM suggest additional new species may exist in this region (Stein & Drazen, 2014; Pyle, Greene & Kosaki, 2016; Sherwood et al., 2020; Alvarado et al., 2022; Sherwood et al., 2022).

Intertidal habitats of the PMNM are known to harbor *Ligia* isopods, a genus of poorly dispersing isopods shown to hold high levels of cryptic diversity (Taiti et al., 2003; Hurtado, Mateos & Santamaria, 2010; Eberl et al., 2013; Santamaria et al., 2013; Raupach et al., 2014; Santamaria, Mateos & Hurtado, 2014; Santamaria et al., 2017; Greenan, Griffiths & Santamaria, 2018; Santamaria, 2019). Currently, nine *Ligia* species are thought to be endemic to the HI: eight coastal species that inhabit rocky intertidal habitats, and a terrestrial species that inhabit terrestrial habitats at elevation in the islands of Kaua‘ i, O‘ ahu and Hawai‘ i. The eight coastal species were formerly recognized as *L. hawaiensis* Dana 1853; however, they were split into these species on the basis of molecular, morphological, and geographic distributional data (Santamaria, 2019). Despite reports of “*L. hawaiensis*” from the islands of Nihoa, Necker, and La Perouse Pinnacle in the PMNM (Taiti & Howarth, 1996), which are located 190 to 675-km from Kaua‘ i, no specimens from these islands were included in any of the molecular studies characterizing *Ligia* from the HI to date (Taiti et al., 2003; Santamaria et al., 2013; Santamaria, 2019). Given the limited dispersal potential exhibited of *Ligia* isopods and the long-term isolation of these oceanic islands, molecular characterizations of these populations are likely to uncover additional cryptic species of *Ligia* in the region.

In this study, we use molecular approaches to characterize *Ligia* isopods from the island of Nihoa, the easternmost island in the PMNM. Doing so, we aim to determine: (a) whether *Ligia* individuals from this highly remote island harbor any unique genetic lineages, (b) if so, what are the phylogenetic relationships of these lineages to other *Ligia* species previously reported from the HI, (c) whether these lineages are divergent enough to be considered a novel species, and if so (d) describe said lineages as a new species. We do so by incorporating phylogenetic reconstructions, and distance- and phylogeny-based molecular species delimitation methods on a multi-locus dataset comprised of all extant *Ligia* species from the Hawaiian Islands and newly collected specimens from Nihoa. Our results indicate *Ligia* from Nihoa represent a highly divergent genetic lineage that is reciprocally monophyletic with all other *Ligia* species from the HI. Given its genetic uniqueness and geographic isolation, we describe this lineage as *Ligia barack* sp. nov. on the basis of molecular characters. The formal description of this cryptic species adds to our understanding of the biodiversity of the PMNM.

## Materials and Methods

### Sample collection

*Ligia* specimens were collected from the splash zone of rocky coastlines of Hanaka‘ ie‘ ie (Adam’s Bay) in Nihoa during April of 2023. All individuals were caught by hand and field-preserved in 70% isopropanol. The collection of specimens from Nihoa was conducted under a permit granted to the Papahanaumokuakea Marine National Monument Co-Trustees, which include the US Fish and Wildlife Service, by the State of Hawai‘ i Board of Land and Natural Resources (Permit Number PMNM-2022-001). Once in the laboratory, specimens were transferred to 70% ethanol. We identified male individuals as members of the *L. hawaiensis* cryptic species complex by inspecting the morphology of the distal process of the endopod of the 2nd pleopod and comparing to previous reports (Taiti et al., 2003). Specimens from Nihoa were then inspected under an AmScope SM-4TZ-144 3.5X-90X Trinocular Zoom Stereo Microscope equipped with a 20 MP imaging system. Drawings of characters were made electronically from images produced in the above mentioned system.

### Molecular laboratory methods

We used Zymo Research’s Quick g-DNA MiniPrep Kit to extract total genomic DNA for six *Ligia* individuals collected in Nihoa. DNA was extracted from 2–3 pereopods per individual using standard protocol instructions. We then used previously published primers and conditions to polymerase chain reaction (PCR) amplify the same four mitochondrial and three nuclear gene fragments used by Santamaria (2019) to conduct a taxonomic revision of *L. hawaiensis*: (a) a 658-bp segment of the Cytochrome Oxidase I gene using primers LCO-1490/HCO-2198 (hereafter COI, primers LCO1490/HCO2198; Folmer et al., 1994), (b) a ∼490-bp segment of the 16S rRNA gene using primers 16Sar/16Sbr (primers 16Sar/16Sbr; Palumbi, 1996), (c) a ∼495-bp segment of the 12S rDNA gene using primers crust-12Sf/crust-12Sr (primers crust-12Sf/crust-12Sr; Podsiadlowski & Bartolomaeus, 2005), (d) a 361-bp fragment of the Cytochrome-b gene using primers 144F and 270R to amplify (hereafter Cyt-b, primers 144F/151F and 270R/272R; Merritt et al., 1998), (e) a ∼1,000-bp segment of the 28S rDNA gene using primers 28SA/28SB (primers 28SA/28SB Whiting, 2002), (f) a 664-bp region of the alpha-subunit of the Sodium Potassium ATPase using primers NaK-forb/NaK-rev2 (hereafter NaK, primers NaK-forb/NaK-rev2; Tsang et al., 2008), and (g) a ∼328-bp fragment of the Histone H3 gene using primers H3AF/H3ARto amplify (primers H3AF/H3AR; Colgan et al., 1998). PCR products were visualized on 1% agarose gels, stained using Apex Safe DNA Gel Stain (Apex Bioresearch Products). Positive amplicons were sequenced at the Arizona Genetics Core.

### Sequence alignment and model testing

Sequences were assembled, edited (*i.e.,* had primers removed), and inspected for evidence indicative of heteroplasmy and/or heterozygosity (*e.g.*, multiple peaks in chromatograms) in CodonCode Aligner v10.0.1. No evidence of heteroplasmy or heterozygosity was observed. Sequences produced in this study were then aligned and added to the aligned dataset produced by Santamaria in 2019 using the “—add” option of the MAFFT webserver (Katoh & Standley, 2013) using standard settings. This dataset includes all currently valid species of *Ligia* endemic to the Hawaiian Islands. Alignments for the three ribosomal genes included in this study (*i.e.,* 28S rDNA, 16S rDNA, and 12S rDNA) were compared to those produced by Santamaria (2019) with poorly aligned sites removed. We inspected alignments of protein coding genes (*i.e.*, COI, Cyt-b, NaK, H3A) and did not observe any evidence suggestive of pseudo-genes such as the presence of early stop codons or indels.

For each gene alignment, we selected the most appropriate model of nucleotide evolution from all available models in jModeltest v2.1 (Darriba et al., 2012) by evaluating their likelihood using a fixed BioNJ-JC tree under the Bayesian information criterion (BIC). Gene alignments were then concatenated using SequenceMatrix v.1.9 (Vaidya, Lohman & Meier, 2011). We used a similar approach as described above to select the most appropriate model of nucleotide evolution for the concatenated alignment. We also selected the most appropriate partition scheme to use in our phylogenetic reconstructions in PartitionFinder v2.1.1 (Lanfear et al., 2016) by evaluating different partitioning combinations of an *a priori* partitioning scheme that consisted of each ribosomal gene as a single partition with protein coding genes separated by gene and codon position. Partitioning schemes were evaluated under the BIC criterion and the following parameters: branch lengths = unlinked; models = all; model selection = BIC; search = greedy. Lastly, we estimated pairwise Kimura-2-parameter (K2P) distances in MEGA v11.0.13 (Kumar, Stecher & Tamura, 2016) for the COI dataset.

### Phylogenetic reconstructions

We conducted phylogenetic reconstructions on the concatenated alignment of all gene fragments under both maximum likelihood and Bayesian inference approaches using two different partitioning approaches: by gene, and as determined by PartitionFinder. Maximum likelihood (ML) searches were conducted in RAxML-NG v1.1.0 (Kozlov et al., 2019) and consisted of 1,000 bootstrap replicates followed by a thorough ML search under the GTR +Γ model run with all other settings as default. Bayesian searches were conducted in MrBayes v3.2.7 (Ronquist & Huelsenbeck, 2003) and consisted of four separate runs consisting each of two chains, run for 20 × 10^6^ generations, sampled every 1,000th generation. All other settings were as default. Bayesian searches were monitored to determine if they had reached and maintained stationarity using the following criteria: (a) stable posterior probability values; (b) high correlation between the split frequencies of independent runs as implemented in AWTY (Nylander et al., 2007); (c) small and stable average standard deviation of the split frequencies of independent runs; (d) potential scale reduction factor close to 1; and (e) an effective sample size (ESS) >200 for the posterior probabilities, as evaluated in Tracer v1.7.2 (Rambaut et al., 2018). For all searches, we calculated majority-rule consensus trees using the SumTrees command of DendroPy v3.10.1 (Sukumaran & Holder, 2010). For Bayesian analyses, samples prior to stationarity were discarded as burn-in.

### Molecular species delimitation analyses (MSDAs)

We implemented both tree- and distance-based species delimitation analyses to determine whether our molecular dataset supports the identification of *Ligia* from Nihoa as a separate species. Tree-based molecular species delimination analyses (MSDAs) were carried out using the Poisson Tree Processes model as implemented in the PTP server (http://species.h-its.org/) and the General Mixed Yule Coalescent model (hereafter GMYC; Fujisawa & Barraclough, 2013). PTP analyses were carried out on all phylogenetic trees produced in RAxML and MrBayes. Settings used were as follows: 500,000 Markov chain Monte Carlo (MCMC) iterations; a burn-in of 0.10; and a thinning value of 100. As GMYC delineations require ultrametric trees as input, we estimated ultrametric trees for the unpartitioned concatenated mitochondrial dataset using BEAST v2.1.3 (Bouckaert et al., 2014) assuming a constant rate of evolution and speciation assuming a Yule process (*i.e.,* constant speciation rate; Yule, 1925; Gernhard, 2008), and under a coalescent model of speciation assuming a constant population size (Kingman, 1982). Both searches were carried out for 50 million generations sampled every 1,000th generation using the most appropriate model of nucleotide evolution. Resulting trees were summarized using the *SumTrees* command with burn-in discarded and with edges set as per the mean-age option. Resulting ultrametric trees were analyzed using the GMYC approach as implemented by the ‘splits’ package (http://r-forge.r-project.org/projects/splits/) in R using default settings.

We conducted distance-based analyses using ASAP (Puillandre et al., 2012) on the COI gene dataset alone after masking ambiguous sites using the ASAP webserver (https://bioinfo.mnhn.fr/abi/public/asap/). ASAP analyses were carried out under the Kimura 2-parameter (K2P) nucleotide evolution model, with all other settings as default. We used KoT (Spöri et al., 2022) to estimate the K/*θ* ratio (Birky Jr et al., 2010; Birky Jr, 2013) between *Ligia* from Nihoa and their most closely related taxa identified by phylogenetic analyses. Analyses were carried on the concatenated dataset assuming a K/*θ* threshold of 4, a value that represents a >95% probability that sister clades have become reciprocally monophyletic (Birky Jr, 2013).

We evaluated the following criteria to determine whether *Ligia* from Nihoa represent a novel species in need of description: (1) did all phylogenetic reconstructions place all Nihoa *Ligia* individuals in a well-supported (bootstrap support (BS) >90%, Bayesian posterior probability (BPP) >95%) monophyletic clade that excluded all other *Ligia* from the Hawaiian Islands; (2) were pairwise COI K2P distances amongst *Ligia* Nihoa specimens <1.0%; (3) do comparisons between *Ligia* from Nihoa and its sister taxon produce a K/*θ*>4 (*i.e.,* 4X rule; Birky Jr, 2013); (4) did most MSDAs separate Nihoa individuals as a putative species; (5) did this putative species exclude all other *Ligia* from the HI. As the answer for all these criteria was affirmative, we herein describe *Ligia barack*, a novel species of *Ligia* from Nihoa. We determined diagnostic nucleotide positions for this novel species using FASTACHAR v0.2.4 (Merckelbach & Borges, 2020) by comparing *L. barack* sp. nov. to all other *Ligia* species endemic to the HI included in the dataset used in this study.

The electronic version of this article in Portable Document Format (PDF) will represent a published work according to the International Commission on Zoological Nomenclature (ICZN), and hence the new names contained in the electronic version are effectively published under that Code from the electronic edition alone. This published work and the nomenclatural acts it contains have been registered in ZooBank, the online registration system for the ICZN. The ZooBank LSIDs (Life Science Identifiers) can be resolved and the associated information viewed through any standard web browser by appending the LSID to the prefix http://zoobank.org/. The LSID for this publication is: urn:lsid:zoobank.org:pub:6CE79D26-19BA-435D-94A8-5A822ADD42B0. The online version of this work is archived and available from the following digital repositories: PeerJ, PubMed Central and CLOCKSS.

## Results

**Figure 1:**
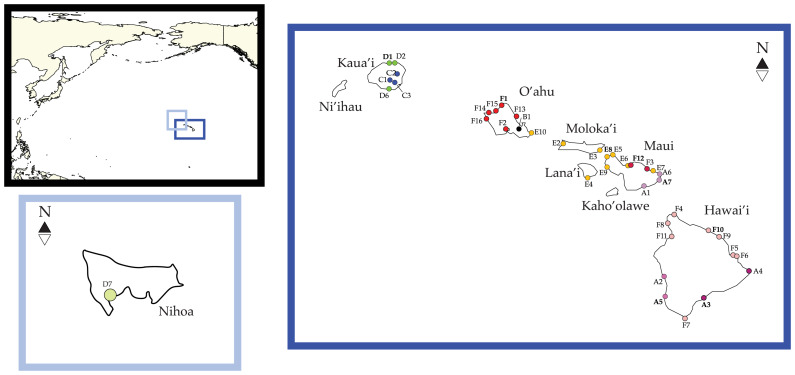
*Ligia* localities included in this study. Labels and colors correspond with other figures and tables in this study and that of Santamaria et al. (2013) and Santamaria (2019). Detailed information for each locality is presented in Table 1. Localities of the suppralittoral *Ligia* included: Kaua‘ i: D1-Kalihiwai Beach, D2-Kauapea Beach, D6-Hoai Bay; D7- Hanaka‘ ie‘ ie (Adam’s Bay); O‘ ahu: E10-Wawamalu Beach Park, F1-Pupukea, F2-Pouhala Marsh, F13-Kahaluu, F14-Kaena Point (North), F15-Kaiaka Bay Beach Park, F16-Kaena Point (South); Moloka‘ i: E2-Papohaku Beach Park, E4-Manele Bay; Lana‘ i: E3-North of Puko‘ o; Maui: A1-Wai‘ Ōpae; A6-Waianapanapa State Park, A7-Koki Beach Park, E5-Poelua Bay, E6-Spreckelsville, E7-Keanae, E8-DT Fleming Beach Park, E9-Hanakao‘ o Park, F3-Honomanu Bay, F12-Baby Beach Spreckelsville Area; Hawai‘ i: A2-Kealakukea Bay, A3-Pu‘ unalu Beach Park, A4-Isaac Hale Beach Park, A5-Miloli Beach Park, F4-Keokea Beach, F5-Onekahakaha Beach Park, F6-Leleiwi Beach, F7-South Point, F8-Kapa‘ a State Park, F9-Kolekole Beach Park, F10-Laupahoehoe Beach Park, F11-Spencer Beach Park. Localities of the terrestrial *L. perkinsi* included are Kaua‘ i: C1-Mt Kahili, C2-Makaleha Mts, C3-Haupu Range; O‘ ahu: B1-Nu‘ uanu Pali. World map is edited from a public domain map produced by Colohisto. Original vector map is available at https://commons.wikimedia.org/wiki/File:BlankMap-World_1990.svg. Map of the Hawaiian Islands is reproduced from Santamaria (2019). Map is available at: https://doi.org/10.7717/peerj.7531/fig-1.

We successfully produced sequences for four mitochondrial and three nuclear genes for six *Ligia* specimens from Nihoa (hereafter *L. barack* sp. nov). Unique haplotypes have been deposited in GenBank and BOLD (see Table 1 for GenBank Accession Numbers). The addition of these sequences to the alignment produced by Santamaria (2019) produced a concatenated dataset 3,996-bp long prior to the removal of poorly aligned positions for the 16S, 12S, and 28S rDNA gene. This final alignment included 196 individuals from 40 localities in the HI including Nihoa (Fig. 1, Table 1). Removal of poorly aligned sites (43, 17, and 49 for the 16S, 12S, and 28S rDNA genes respectively) produced a final alignment 3,887-bp long containing 543 parsimony informative sites (COI = 185; Cyt-b: 120; 12S rDNA = 99; 16S rDNA = 91; 28S rDNA = 39; NaK = 6; H3A = 3). An annotated alignment is provided as Dataset S1.

**Table 1:** Localities included in the study, with corresponding number of individuals included, GenBank accession numbers, and geographic information.

Loc. Label	Locality Name	New Loc.	# inds.	COI Acc. No	16S rDNA Acc, No.	12S rDNA. Acc. No.	Cytb Acc. No.	28S rDNA Acc. No.	NaK Acc. No.	H3A Acc. No.	Latitude	Longitude
A1	Wai‘Ōpae Maui	NO	2	MK034488	MK032502 KF546549	MK032601 KF546573	MK034572 KF546718	N/A	N/A	MK034658	20°37′29.20″N	156°12′34.10″W
A2	Kealakukea Bay Hawai‘ i	NO	6	MK034474 MK034475 MK034476 MK034477 KF546627	MK032515 MK032516 MK032517 MK032518 MK032519	MK032608 MK032609 KF546574		MK940873 MK940874	KF546594	MK034663	19°28′32.88″N	155°55′11.04″W
A3	Pu‘ unalu Beach Park Hawai‘ i	NO	5	MK034513 MK034514 KF546628	MK032564 MK032565 MK032566 MK032567 KF546551	MK032627 MK032628 KF546576	MK034582 MK034583 KF546716	MK940887 KF546701	KF546593	MK034677	19°08′00.60″N	155°30′18.30″W
A4	Isaac Hale Beach Park Hawai‘ i	NO	6	N/A	MK032568 MK032569 MK032570 MK032571 MK032572 KF546550	MK032629 MK032630 KF546575	MK034584 MK034585 KF546717	MK940888 KF546702	MK034645 MK034646 KF546586	MK034678 MK034679	19°27′26.82″N	154°50′31.68″W
A5	Miloli Beach Park Hawai‘ i	NO	5	MK034478 MK034479 MK034480 MK034481 MK034482	MK032554 MK032555 MK032556 MK032557 MK032558	MK032623 MK032624	MK034567 MK034568 MK034569	MK940885 MK940886	MK034642 MK034643	MK034675	19°10′58.10″N	155°54′25.10″W
A6	Waianapanapa State Park Maui	NO	5	N/A	MK032492 MK032493 MK032494 MK032495 MK032496	MK032596 MK032597	MK034570 MK034571	MK940866	MK034605	MK034654 MK034655	20°47′21.80″N	156°00′07.90″W
A7	Koki Beach Park Maui	NO	5	MK034483 MK034484 MK034485 MK034486 MK034487	MK032497 MK032498 MK032499 MK032500 MK032501	MK032598 MK032599 MK032600		MK940867	MK034606 MK034607 MK034608 MK034609 MK034610	MK034656 MK034657	20°43′41.62″N	155°59′06.71″W
B1	Nu‘ uanu Pali O‘ ahu	NO	1	KF546661	KF546548	KF546572	KF546719	N/A	N/A	N/A	N/A	N/A
C1	Mt Kahili Kaua‘ i	NO	1	KF546660	KF546546	KF546578	N/A	N/A	N/A	N/A	N/A	N/A
C2	Makaleha Mts Kaua‘ i	NO	1	KF546659	KF546545	KF546577	KF546723	N/A	N/A	N/A	N/A	N/A
C3	Haupu Range Kaua‘ i	NO	1	KF546655	KF546547	KF546579	KF546722	KF546683	KF546592	N/A	N/A	N/A
D1	Kalihiwai Beach Kaua‘ i	NO	14	MK034540 MK034541 MK034542 MK034543 MK034544 KF546598 KF546599 KF546600 KF546601 KF546602 KF546603 KF546604 KF546605 KF546606	MK032544 MK032545 MK032546 MK032547 MK032548 KF546544	MK032619 MK032620 KF546571	MK034593 MK034594 KF546721	MK940882 MK940883 KF546686 KF546687 KF546688 KF546689 KF546690	MK034635 MK034636 MK034637 MK034638 MK034639 KF546585	MK034672 MK034673	22°13′05.30″N	159°25′31.15″W
D2	Kauapea Beach Kaua‘ i	NO	1	KF546656	KF546543	KF546570	KF546720	N/A	N/A	N/A	N/A	N/A
D6	Hoai Bay Kaua‘ i	NO	5	MK034545 MK034546 MK034547 MK034548 MK034549	MK032549 MK032550 MK032551 MK032552 MK032553	MK032621 MK032622	MK034595 MK034596	MK940884	MK034640 MK034641	MK034674	21°52′51.93″N	159°28′25.01″W
D7	Hanaka‘ ie‘ ie (Adam’s Bay), Nihoa	YES	6	PP851829 PP851830 PP851831 PP851832 PP851833 PP851834	PP852382 PP852383 PP852384 PP852385 PP852386 PP852387	PP852388 PP852389 PP852390 PP852391 PP852392 PP852393	PP856001 PP856002 PP856003 PP856004 PP856005 PP856006	PP852394 PP852395 PP852396 PP852397 PP852398 PP852399	PP856007 PP856008 PP856009 PP856010 PP856011 PP856012	PP861092 PP861093 PP861094 PP861095 PP861096 PP861097	23°03′30.30″N	161°55′27.60″W
E2	Papohaku Beach Park Moloka‘i	NO	1	KF546607	KF546542	KF546569	KF546715	N/A	N/A	N/A	21°10′46.56″N	157°15′5.88″W
E3	North of Puko‘ o Lana‘i	NO	9	KF546608 KF546609 KF546610 KF546611 KF546612 KF546613 KF546614 KF546615 KF546616	KF546540	KF546565	KF546713	KF546696 KF546697 KF546698 KF546700	KF546587	N/A	21°06′06.84″N	156°45′06.66″W
E4	Manele Bay Moloka‘i	NO	7	KF546643 KF546644 KF546645 KF546646 KF546647 KF546648 KF546649	KF546538	KF546564	N/A	KF546677 KF546678 KF546679 KF546680 KF546681 KF546682	KF546589	N/A	20°44′37.37″N	156°53′12.47″W
E5	Poelua Bay Maui	NO	1	KF546657	KF546532	KF546566	KF546710	N/A	N/A	N/A	N/A	N/A
E6	Spreckelsville Maui	NO	8	KF546595 KF546596 KF546597 KF546650 KF546651 KF546652 KF546653 KF546654	KF546539	KF546567	KF546712	KF546691 KF546692 KF546693 KF546694 KF546695	KF546590	N/A	20°54′31.38″N	156°24′40.26″W
E7	Keanae Maui	NO	6	KF546658	MK032487 MK032488 MK032489 MK032490 MK032491 KF546537	MK032594 MK032595 KF546568	MK034597 MK034598 KF546714	MK940865	N/A	MK034652 MK034653	N/A	N/A
E8	DT Fleming Beach Park Maui	NO	2	MK034550 MK034551	MK032503 MK032504	MK032602 MK032603	MK034599 MK034600	MK940868 MK940869	MK034611 MK034612	MK034659 MK034660	21°00′20.82″N	156°38′58.43″W
E9	Hanakao‘ o Park Maui	NO	5	MK034552 MK034553 MK034554 MK034555 MK034556	MK032505 MK032506 MK032507 MK032508 MK032509	MK032604 MK032605	MK034601 MK034602	MK940870 MK940871	MK034613 MK034614 MK034615 MK034616	N/A	20°54′34.10″N	156°41′19.03″W
E10	Wawamalu Beach Park O‘ ahu	NO	5	MK034557 MK034558 MK034559 MK034560 MK034561	MK032535 MK032536 MK032537 MK032538 MK032534	MK032616 MK032617	MK034603 MK034604	MK940879	MK034628 MK034629 MK034630 MK034631 MK034632	MK034669	21°17′12.51″N	157°40′07.66″W
F1	Pupukea O‘ ahu	NO	16	MK034494 MK034495 MK034496 MK034497 KF546617 KF546618 KF546619 KF546620 KF546621 KF546622 KF546623 KF546624 KF546625 KF546626	MK032520 MK032521 MK032522 MK032523 KF546533 KF546531	MK032610 MK032611 KF546562	MK034575 MK034591 KF546709	KF546667 KF546668 KF546669 KF546670 KF546671	MK034621 MK034622 MK034623 KF546591	MK034664 MK034665	21°38′59.70″N	158°03′45.48″W
F2	Pouhala Marsh O‘ ahu	NO	1	N/A	KF546532	N/A	KF546710	N/A	N/A	N/A	N/A	N/A
F3	Honomanu Bay Maui	NO	1	N/A	KF546530	KF546563	KF546708	N/A	N/A	N/A	N/A	N/A
F4	Keokea Beach Hawai‘ i	NO	1	N/A	KF546529	KF546558	KF546703	N/A	N/A	N/A	N/A	N/A
F5	Onekahakaha Beach Park Hawai‘ i	NO	19	MK034520 MK034521 MK034522 MK034523 MK034524 KF546629 KF546630 KF546631 KF546632 KF546633 KF546634 KF546635 KF546636 KF546637 KF546638 KF546639 KF546640 KF546641 KF546642	MK032573 MK032574 MK032575 MK032576 KF546534	MK032631 MK032632 KF546561	MK034588 KF546705	KF546672 KF546673 KF546674 KF546675 KF546676	KF546588	MK034680 MK034681	19°44′16.05″N	155°02′20.15″W
F6	Leleiwi Beach Hawai‘ i	NO	1		KF546535	KF546560	KF546706	N/A	N/A	N/A	N/A	N/A
F7	South Point Hawai‘ i	NO	6	MK034515 MK034516 MK034517 MK034518 MK034519	MK032559 MK032560 MK032561 MK032562 MK032563 KF546536	MK032625 MK032626 KF546559	MK034586 MK034587 KF546707	N/A	MK034644	MK034676		
F8	Kapa‘ a State Park Hawai‘ i	NO	1		KF546528	KF546557	KF546704	N/A	N/A	N/A	20°12′11.52″N	155°54′6.66″W
F9	Kolekole Beach Park Hawai‘ i	NO	5	MK034525 MK034526 MK034527 MK034528 MK034529	MK032577 MK032578 MK032579 MK032580 MK032581	MK032633 MK032634	MK034589 MK034590	MK940891 MK940892	MK034647	N/A	19°52′58.80″N	155°07′07.60″W
F10	Laupahoehoe Beach Park Hawai‘ i	NO	5	MK034530 MK034531 MK034532 MK034533 MK034534	MK032582 MK032583 MK032584	MK032635 MK032636	MK034591	MK940893 MK940894 MK940895 MK940896	MK034648	MK034682 MK034683	19°59′36.60″N	155°14′24.01″W
F11	Spencer Beach Park Hawai‘ i	NO	5	MK034535 MK034536 MK034537 MK034538 MK034539	MK032585 MK032586 MK032587 MK032588 MK032589	MK032637 MK032638	MK034592	N/A	MK034649 MK034650 MK034651	MK034684 MK034685	20°01′22.41″N	155°49′21.50″W
F12	Baby Beach Maui	NO	7	MK034562 MK034563 MK034564 MK034565 MK034566	MK032482 MK032483 MK032484 MK032485 MK032486	MK032592 MK032593	N/A	MK940864	N/A	N/A	20°54′45.09″N	156°24′16.01″W
F13	Kahaluu O‘ ahu	NO	5	MK034489 MK034490 MK034491 MK034492 MK034493	MK032510 MK032511 MK032512 MK032513 MK032514	MK032606 MK032607	MK034573 MK034574	MK940872	MK034617 MK034618 MK034619 MK034620	MK034661 MK034662	21°28′17.81″N	157°50′40.65″W
F14	Kaena Point (North) O‘ ahu	NO	5	MK034498 MK034499 MK034500 MK034501 MK034502	MK032524 MK032525 MK032526 MK032527 MK032528	MK032612 MK032613	MK034576 MK034577	MK940875 MK940876	MK034624 MK034625	MK034666 MK034667	21°34′47.46″N	158°14′15.43″W
F15	Kaiaka Bay Beach Park O‘ ahu	NO	5	MK034503 MK034504 MK034505 MK034506 MK034507	MK032529 MK032530 MK032531 MK032532 MK032533	MK032614 MK032615	MK034578 MK034579	MK940877 MK940878	MK034626 MK034627	MK034668	21°35′20.62″N	158°07′03.42″W
F16	Kaena Point (South) O‘ ahu	NO	5	MK034508 MK034509 MK034510 MK034511 MK034512	MK032590 MK032539 MK032540 MK032541 MK032542 MK032543	MK032618	MK034580 MK034581	MK940880 MK940881	MK034633 MK034634	MK034670 MK034671	21°33′21.21″N	158°14′54.88″W

**Figure 2:**
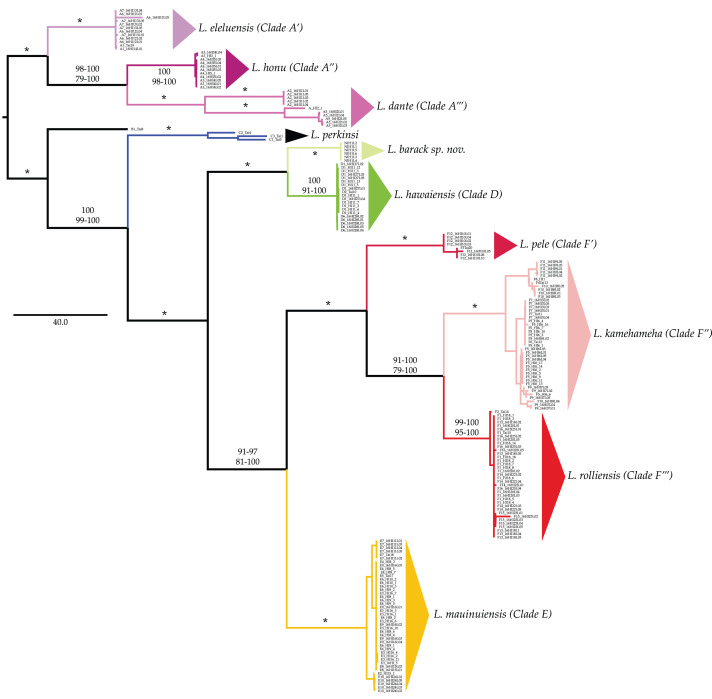
Majority rule consensus tree of bootstrap replicates produced by analyzing the concatenated mitochondrial and nuclear dataset of *Ligia* from the Hawaiian Islands in RAxML under the GTR +Γ under a “by gene” partitioning scheme. Branches and clades are colored as per Santamaria et al. (2013) and Santamaria (2019). Values by nodes correspond with bootstrap support values observed in RAxML analyses (above) and posterior probabilities produced in MrBayes analyses (below). Asterisks (*) denote 100% support across all analyses.

All phylogenetic reconstructions completed in this study were similar to those reported by Santamaria et al. (2013) and Santamaria (2019), with the exception of *L. barack* sp. nov individuals (Fig. 2). These individuals were placed in a well-supported clade (BS = 100; PP = 100, Fig. 2) that excluded individuals from all other *Ligia* species endemic to the HI. Our phylogenetic reconstructions identified four highly divergent and reciprocally monophyletic lineages consisting solely of coastal *Ligia* endemic to the Hawaiian Islands: (a) *Clade A* (lavenders and purples in all figures; BS = 100; posterior probability (PP) = 100) which consisted of all *L. dante* (A2, A5 in Hawai‘ i), *L. honu* (A3–4 in Hawai‘ i) and *L. eleluensis* (A1, A6–7 in Maui) individuals; (b) *Clade D* (green in all figures; BS =100; PP =100) which included all *L. hawaiensis* individuals from Kaua‘ i (D1–2, D6) as well as *L. barack* sp. nov. (D7); (c) *Clade E* (oranges and yellows in all figures; BS = 100; PP = 100) consisting of all *L. mauinuiensis* individuals from O‘ ahu (E10), Moloka‘ i (E2, E3), Lana‘ i (E4), and Maui (E5–E9); and lastly (d) *Clade F* (reds in all figures; BS = 100; PP = 100) which included all *L. kamehameha* (F4–F11 in Hawai‘ i), *L. rolliensis* (F1–2 and F13–16 in O‘ ahu), and *L. pele* (F3, F12 in Maui) individuals. We also observed two lineages consisting of individuals of the terrestrial *L. perkinsi*: (a) *Clade B* (black in all figures) from O‘ ahu (B1), and (b) *Clade C* (blue in all figures; BS = 100; PP = 100) from Kaua‘ i (C1–3).

Clades *D*, *E*, and *F* were placed in a well-supported monophyletic group (BS = 100; PP = 100) with clades *E* and *F* identified as each other’s sister clade (BS = 81–100; PP = 91–97). The sister to the “*D* + *E* + *F*” clade was *Clade C* (BS = 99–100; PP = 100), which consisted of the terrestrial *L. perkinsi* from Kaua‘ i. *Clade B*, consisting of the terrestrial *L. perkinsi* from O‘ ahu, was identified as the sister clade to the large monophyletic group consisting of clades *C*, *D*, *E*, and *F* (BS = 100; PP = 100). The most basal group was *Clade A*, which consisted of coastal *Ligia* species from the islands of Maui and Hawai‘ i.

COI K2P distances between *Ligia* species from HI ranged between 3.0–17.8%, with comparisons between *L. barack* sp. nov and other species in the region ranging between 3.0–17.8% (Table 2). Genetic diversities produced when comparing *L. barack* sp. nov were low, ranging between 0.0–0.3% (Table 2).

**Table 2:** Estimates of evolutionary divergence, as measured by Kimura 2-parameter distances, for *Ligia barack* and other *Ligia* species from the Hawaiian Islands.

	*L. barack*	*L. dante*	*L. honu*	*L. eleluensis*	*L. perkinsi* (O‘ ahu)	*L. perkinsi* (Kaua‘ i)	*L. hawaiiensis*	*L. mauinuiensis*	*L. rolliensis*	*L. kamehameha*
*L. barack*	0.0–0.3 (0.2)									
*L. dante*	13.9–15.2 (14.5)	0.0-4.6 (2.4)								
*L. honu*	14.9–15.1 (15)	5.8–7.5 (6.8)	N/A							
*L. eleluensis*	17–17.8 (17.4)	9.7–11.2 (10.7)	10.9–11.3 (11.0)	0.0–0.9 (0.5)						
*L. perkinsi* (O‘ ahu)	14.8–15.0 (14.9)	14.2–15.0 (14.5)	15.1–15.1 (15.1)	14.3–14.8 (14.6)	N/A					
*L. perkinsi* (Kaua‘ i)	15.8–16.9 (16.4)	11.9–14.3 (13.2)	13.7–15.1 (14.1)	12.5–13.9 (13.2)	14.5–15.3 (14.9)	1.0–2.7 (1.9)				
*L. hawaiiensis*	3.0–4.0 (3.5)	12.8–15.4 (14.4)	15.0–16.9 (16.4)	14.7–16.4 (15.8)	14.8–15.8 (15.5)	13.8–15.6 (15.1)	0.0–2.2 (0.9)			
*L. mauinuiensis*	11.5–13.2 (12.5)	13.6–15.9 (14.9)	14.5–15.3 (15.0)	13.9–16.4 (15.3)	16.0–16.6 (16.4)	12.5–14.2 (13.0)	10.3–12.7 (11.5)	0.0–2.4 (0.7)		
*L. rolliensis*	12.5–14.3 (13.6)	14.0–16.6 (15.3)	14.9–16.2 (15.3)	13.4–16.2 (14.5)	13.7–14.7 (14.1)	12.9–16.1 (13.9)	12.5–14.8 (13.6)	10.9–12.8 (11.6)	0.0–7.1 (1.9)	
*L. kamehameha*	12.3–15.4 (13.5)	13.8–16.4 (14.8)	13.6–15.5 (14.6)	13.8–15.4 (14.6)	12.6–13.9 (13.2)	11.1–15.0 (13.2)	11.6–14.6 (12.9)	8.7–13.1 (10.6)	4.0–8.7 (5.3)	0.0–5.4 (2.5)

Molecular species delimitations consistently identified *L. barack* sp. nov as a separate and distinct species from other *Ligia* species endemic to the HI. ASAP analyses of the COI dataset placed all Nihoa specimens in a separate putative species containing no *Ligia* from other localities in nine of the ten best partitions produced by ASAP, with only the ninth best supported partition (*p*-value rank = 4; W rank = 18; threshold distance = 0.040249) grouping Nihoa *Ligia* with *L. hawaiensis* individuals (Kaua‘ i). All tree-based MSDAs carried out in PTP, bPTP, and GMYC recognized *L. barack* sp. nov as a separate species. Lastly, comparisons between this newly described species and *L. hawaienesis*, its sister taxon, in KoT produced a K/*θ* ratio of 9.912.

## Taxonomy

**Figure 3:**
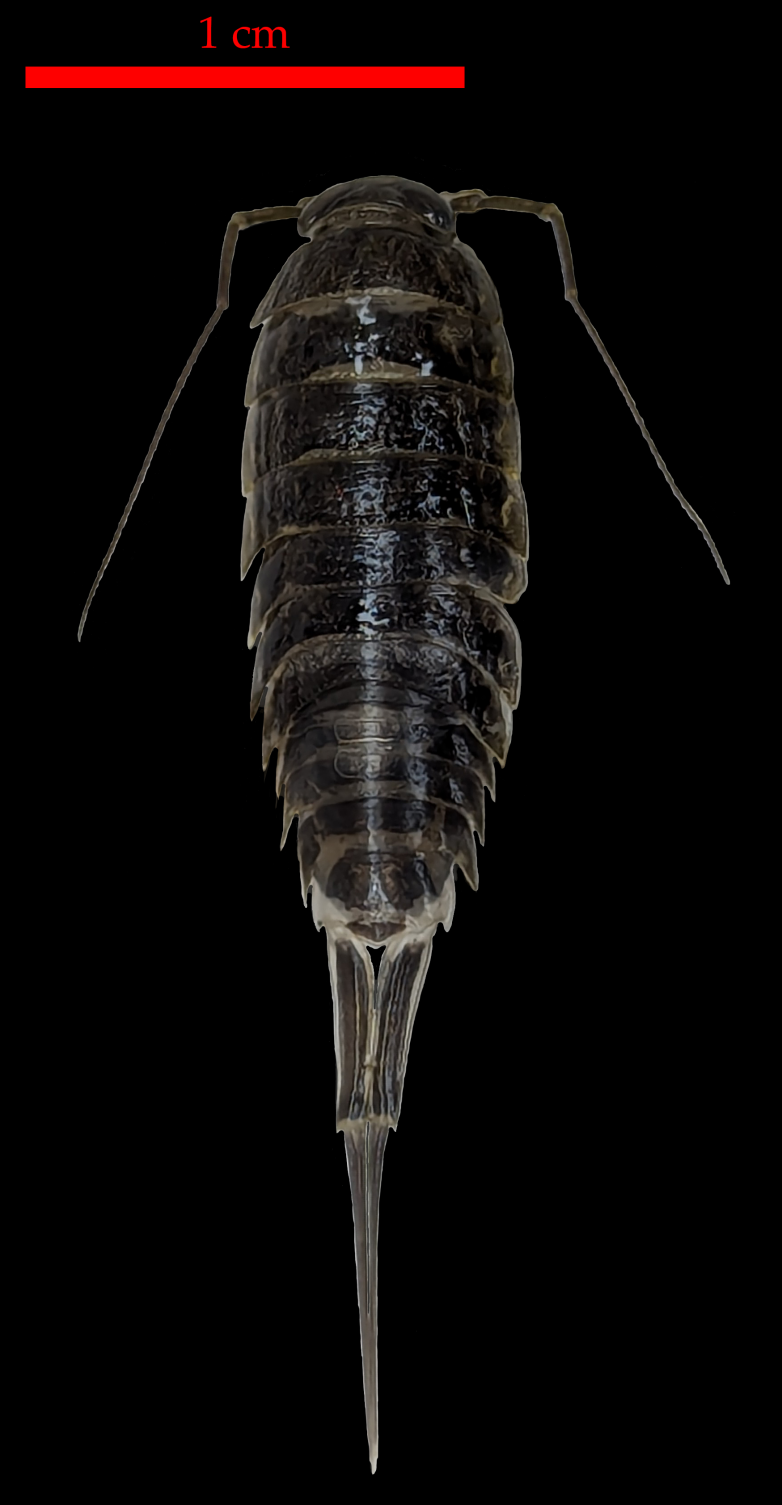
Holotype of *Ligia barack*, a new species from Nihoa. Holotype shown in this picture is deposited at the Florida Museum of Natural History (UFID 72496).

**Figure 4:**
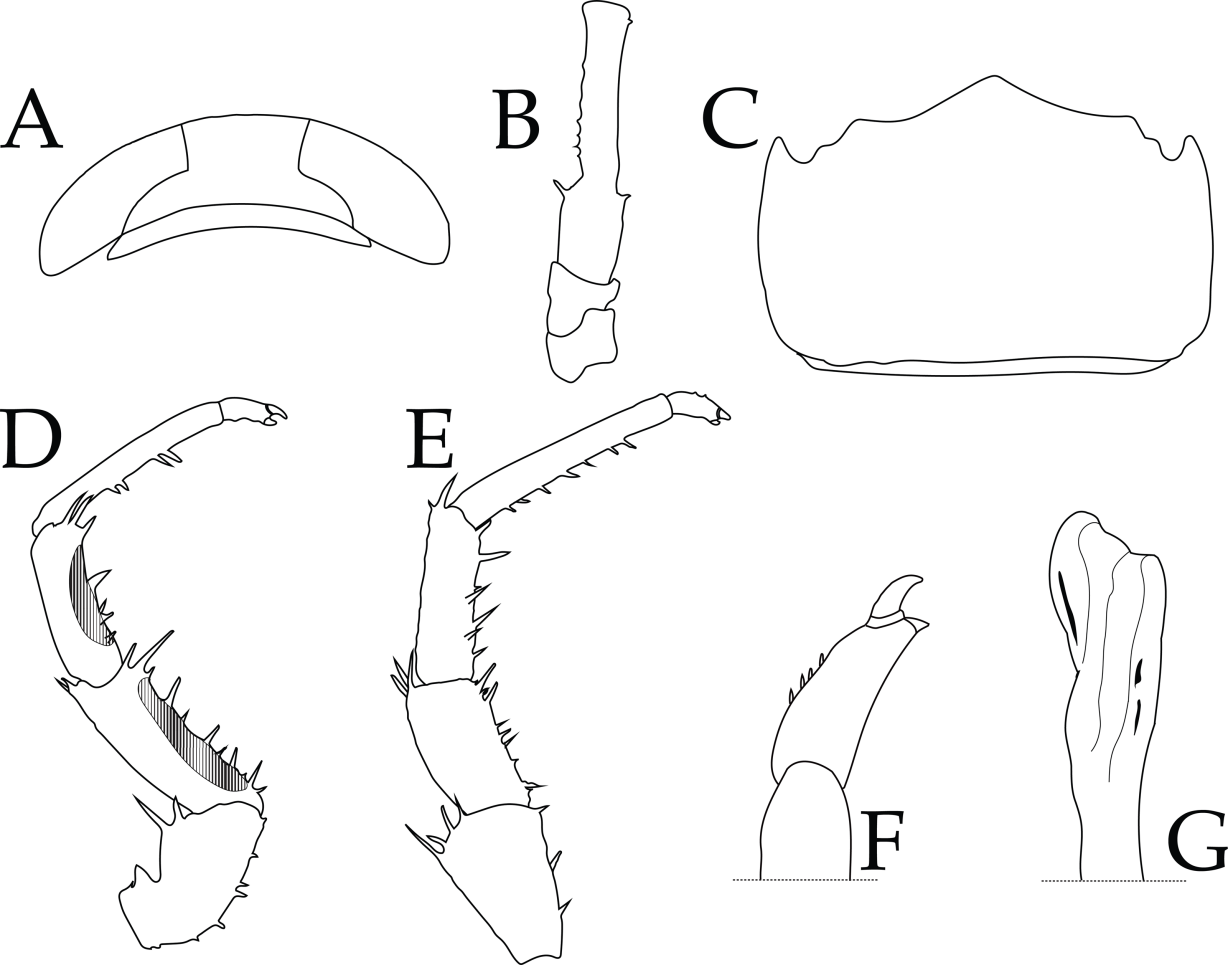
*Ligia barack* nov. sp. holotype (UFID 72496). (A) Cephalon; (B) peduncles 1–3 of antenna; (C) Pleotelson, dorsal view; (D) Pereopod 1; (E) Pereopod 6; (F) Closeup of dactylus of Pereopod 6; (G) apex of appendix masculina of second pleopod.

Based on the long-term and geographical isolation for Nihoa, morphological comparisons, results of phylogenetic reconstructions and MSDAs, COI K2P pairwise distances reported herein, and K/*θ* ratio between it and its sister taxon, we describe *Ligia barack* sp. nov., a new species of *Ligia* from Nihoa. A holotype and three paratypes were deposited at the Florida Museum of Natural History (FLMNH) in Gainesville, FL, USA. We describe *L. barack* sp. nov. primarily using molecular characters and some diagnostic morphological characters. We include a broad description of the holotype that covers traits evaluated by previous authors (Taiti et al., 2003; Santamaria et al., 2013; Santamaria, 2019), a photograph of the holotype of *L. barack* (Fig. 3), and illustrations of diagnostic features of this species (Fig. 4). Other traits not mentioned below (*e.g.*, pereopods 2–6) are as described and/or illustrated by Taiti et al. (2003), Taiti, Ferrara & Kwon (1992), and Jackson (1933).

***Ligia barack*** nov. sp.

LSID: urn:lsid:zoobank.org:act:558494AB-37D7-47BA-BA54-4E532D7585C6.

BOLD BINs: AFQ9578.

Materials examined: six individuals from the island of Nihoa (D7). Both males and females were included. The holotype (♂; UFID 72496; Fig. 3), and three paratypes (♀♀♀; UFID 72497-72499) from the type locality have been deposited at the Florida Museum of Natural History (FLMNH) in Gainesville, FL, USA.

Type locality: Hanaka‘ ie‘ ie (Adam’s Bay), Nihoa, Hawai‘ i, USA. (D7; 23°03′30.3″N 161° 55′27.6″W).

Description of holotype: male individual that is 17.8 mm long and 6.7 mm wide at the widest point of the pereionite 4 (body length to width ratio of ∼2.7; Fig. 2). Eyes are large (eye length is ∼0.5 greatest width of cephalon) and closely spaced (inter-eye distance ∼0.5 times eye length) (Fig. 4A). Posterolateral processes of the pereionite 7 extend ∼$ \frac{1}{3} $ length of the pleonite 3. Antennae extends just past midbody, being about ∼0.6 times the total body length. Article 3 of the peduncle of the antenna is about 1.5X length of the peduncle article 2, with robust setae on either side of the distal end (Fig. 4B). Pleotelson shape similar to that of other *Ligia* in the Hawaiian Islands; however, the lateral posterior points are nearly parallel to the interior lateral posterior points (Fig. 4C). First three pereopods have papillar fields present, with those in the carpus and merus of the first pereopod present in over half of pereopod segment (*e.g.*, pereopod 1 illustrated in Fig. 4D). The dactylus of the sixth and seventh pereopod have four small robust setae on the tergal margin (Fig. 4F). Apex of the appendix masculina in the endopod of the second pleopod is enlarged and slightly bilobed with distal end rounded (Fig. 4G).

The holotype is deposited in the FLMNH under UFID 72496. GenBank Accession numbers for sequences obtained from the holotype are: PP851829 (COI); PP852382 (16S rDNA); PP852387 (12S rDNA); PP856001 (Cyt-b); PP852394; (28S rDNA); PP856007 (NaK); and PP861092 (H3A).

Remarks: the herein described *L. barack* sp. nov can be distinguished from other coastal *Ligia* species endemic to the Hawaiian Islands by the absence of a tuft of very long thin setae on the tergal margin of the dactylus of the sixth and seventh pereopods (Figs. 4E–4F; see Fig. 4 in Taiti et al., 2003). The appendix masculina of the endopod of the second pleopod also differs between *L. barack* sp. nov and other species in the region (Taiti et al., 2003). The appendix masculina of *L. barack* is slightly bilobed with the distal end being rounded, which contrasts with the obliquely truncate morphology seen in coastal *Ligia* from the Hawaiian Islands (see Fig. 4G; contrast with Fig. 6 in Taiti et al., 2003). Lastly, *L. barack* exhibits slight differences in eye shape and size. This species has large eyes (eye length is ∼0.5 greatest width of cephalon) which is similar to other coastal *Ligia* species endemic to the Hawaiian Islands; however, the eyes appear to be the most distantly spaced of all these species. The inter-eye distance of *L. barack* is about ∼0.5 times the eye length, while the smallest ratio for other nearby species is ∼0.7 (seen in *L. honu*, *L. hawaiiensis*, *L. kamehameha*, *L, mauiniensis, L. rolliensis*; Santamaria, 2019). Lastly *L. barack* can also be distinguished using molecular characters listed below.

Diagnostic molecular characters:

*COI:* 1-C; 31-A; 94-C; 526-C

*16S:* 288(316)-T.

*Cyt-b:* 181-G; 223-C; 262-G; 265-G; 354-G

*12S:* 380(398)-G.

Distribution: rocky intertidal habitats of Nihoa.

Hawaiian common name: Pokipoki o Hanaka‘ ie‘ ie. *Pokipoki* is the Hawaiian name for terrestrial isopods and similar creatures inhabiting aquatic and terrestrial habitats. Meanwhile, *Hanaka‘ ie‘ ie* refers to the traditional name for Adam’s Bay of Nihoa Island. Thus, this name broadly translates to “the isopod from Adam’s Bay of Nihoa Island.”

Etymology: this species is named after Barack H. Obama, the former President of the United States of America, who was born in the island of O‘ ahu and who is responsible for the expansion of the Papahānaumokuākea Marine National Monument to its current size.

## Discussion

The Hawaiian Islands (HI) were previously thought to harbor a single endemic coastal *Ligia* species: *Ligia hawaiensis*. This species, first described by Dana (1853), was determined to represent a cryptic species complex composed of allopatric species with distributional ranges largely limited to rift zones within a single island, single islands, or previously connected islands (Santamaria, 2019). Despite previous reports of *L. hawaiensis* from the remote and older islands found in the Papahānaumokuākea Marine National Monument (Taiti & Howarth, 1996), none of the molecular studies conducted on Hawaiian *Ligia* to date have included populations from these islands. This has left unanswered whether *Ligia* populations from the older and highly remote islands in the PMNM harbor highly divergent genetic lineages and/or novel species in need of description. By using similar molecular approaches to those used by Santamaria (2019) to describe highly genetically divergent yet largely morphologically cryptic lineages of *Ligia* in the HI as new species, we herein describe *L. barack* sp. nov from Nihoa.

Our molecular characterizations of *Ligia* individuals collected in Nihoa show this population to be highly divergent and isolated from other *Ligia* lineages and species found in the HI. We observed no sharing of haplotypes between *L. barack* sp. nov individuals and other *Ligia* populations in the HI at any of the four mitochondrial genes studied (*e.g.*, COI, Cyt-b, 16S and 12S rDNA). Instead, Nihoa specimens harbored unique and private haplotypes that form a well-supported monophyletic group that excludes all other *Ligia* species from the HI and that are highly divergent from other ones found to date in Hawaiian *Ligia*. COI K2P divergences between Nihoa *Ligia* and other *Ligia* species from Hawaii ranged from 3.0–17.8%, values that are similar to other amongst species comparisons (Table 2). Meanwhile, within species COI K2P divergences amongst *L. barack* sp. nov individuals ranged from 0.0–0.7%. Not surprisingly, the K/*θ* ratio between *L. barack* sp. nov and its sister taxon (*L. hawaiensis*; K/*θ* = 9.912), greatly exceeds the K/*θ* ratio of 4 at which there is a 95% probability that two separate species are being compared (Rosenberg, 2003).

The phylogenetic placement of *L. barack* sp. nov is of interest, as our analyses recovered with high support both the monophyly of this newly described species and its sister relationship to *L. hawaiensis* (Fig. 4). The latter is a coastal species of *Ligia* whose distributional range is thought to be limited to the island of Kaua‘ i, the closest island to Nihoa. These islands are separated by ∼240 km of open ocean and have never been connected. This suggests that oceanic dispersal led to the colonization of these islands by *Ligia*. Nihoa’s older age (7.5 My) suggests the ancestor to *L. hawaiensis* in Kaua‘ i may have originated from Nihoa; however, additional work is necessary to establish the origins of these species as back-dispersals appear to have occurred in *Ligia* from the HI.

Despite consisting of the *Ligia* from the two oldest islands in our analyses (Kaua‘ i: 5 My, Nihoa 7.5 My), the monophyletic group consisting of *L. hawaiensis* + *L. barack* sp. nov clade was not found in a basal position in any of our analyses (Fig. 3). Instead, the most basal clade in all analyses was one comprised of *Ligia* species found in Maui and Hawai‘ i, the two youngest islands in the archipelago (<1.5 My). These findings are consistent with previous studies of *Ligia* from the HI (Santamaria et al., 2013; Santamaria, 2019) and suggest that the evolution of *Ligia* in the region have been shaped by colonization, extinction, and back-dispersal events.

Our description of *L. barack* sp. nov from the island of Nihoa underscores the importance of molecular approaches in conservation efforts in the PNMN. Future studies of *Ligia* from other islands in the PNMN are likely to uncover additional highly divergent genetic lineages likely representing new species in need of description. These studies may also help further elucidate the evolutionary history of *Ligia* in the HI. Meanwhile, molecular characterizations of other poorly dispersing organisms may similarly uncover new species or genetic lineages in other taxa and thus increase our understanding of the biodiversity of these highly remote and isolated islands. Molecular tools may also aid in the monitoring of the spread of alien species, a critical threat to the fauna and flora of the PMNM (DeFelice et al., 1998; Selkoe, Halpern & Toonen, 2008). *Ligia exotica* has been shown to occur in Midway Atoll, an island within the PMNM (Santamaria et al., 2022). This species of Asian origin known to have been introduced to manmade coastal habitats around the world and is a potential competitor to endemic coastal *Ligia* (Hurtado et al., 2018). The use of genetic tools such as COI barcoding and eDNA may be useful to monitor the presence of this species in other regions of the PMNM without extensive field-work.

## Conclusion

The use of both mitochondrial and nuclear gene fragments to characterize *Ligia* isopods from Nihoa uncovered a highly divergent lineage of Hawaiian *Ligia* not previously reported from other localities in the HI. Phylogenetic and species delimitation approaches provide evidence that this lineage represents a new species of *Ligia*, which we describe as *Ligia barack* sp. nov. To our knowledge, this species is the first intertidal crustacean that is described from and likely solely endemic to the island of Nihoa. This discovery underscores the unique biodiversity of the PMNM and the need for additional studies of poorly dispersing taxa within it. Our findings also further provide evidence of *Ligia* isopods as an example of *in-situ* speciation of a Hawaiian marine animal.

##  Supplemental Information

10.7717/peerj.19373/supp-1 Supplemental Information 1 Concatenated alignment used for phylogenetic analyses and MSDAs

## Additional Information and Declarations

### Field Study Permissions

The following information was supplied relating to field study approvals (i.e., approving body and any reference numbers): The collection of specimens from Nihoa was conducted under a permit granted to the Papahanaumokuakea Marine National Monument Co-Trustees, which include the U.S. Fish and Wildlife Service, by the State of Hawai‘ i Board of Land and Natural Resources (Permit Number PMNM-2022-001).

### DNA Deposition

The following information was supplied regarding the deposition of DNA sequences: The sequences obtained from the holotype are available at GenBank: PP851829 (COI); PP852382 (16S rDNA); PP852388 (12S rDNA); PP856001 (Cyt-b); PP852394; (28S rDNA); PP856007 (NaK); and PP861092 (H3A).

### Data Availability

The following information was supplied regarding data availability: The aligned dataset used for all analyses in this study is available in the Supplementary File.

### New Species Registration

The following information was supplied regarding the registration of a newly described species: Publication LSID: urn:lsid:zoobank.org:pub:6CE79D26-19BA-435D-94A8-5A822ADD 42B0 Species name: urn:lsid:zoobank.org:act:558494AB-37D7-47BA-BA54-4E532D7585C6.
